# Measurement of Cytokines and Adhesion Molecules in the First 72 Hours after Severe Trauma: Association with Severity and Outcome

**DOI:** 10.1155/2015/747036

**Published:** 2015-03-11

**Authors:** António Sousa, Frederico Raposo, Sara Fonseca, Luís Valente, Filipe Duarte, Moura Gonçalves, Diana Tuna, José-Artur Paiva

**Affiliations:** ^1^Centro Hospitalar de São João (CHSJ), Porto, Portugal; ^2^Orthopaedic Department and Emergency and Intensive Care Department, Alameda Professor Hernani Monteiro, 4200-319 Porto, Portugal; ^3^CHSJ, Orthopaedic Department and Emergency and Intensive Care Department, Alameda Professor Hernani Monteiro, 4200-319 Porto, Portugal; ^4^CHSJ, Anesthesiology Department, Alameda Professor Hernani Monteiro, 4200-319 Porto, Portugal; ^5^CHSJ, Orthopaedic Department, Alameda Professor Hernani Monteiro, 4200-319 Porto, Portugal; ^6^Centro Hospitalar São João, Orthopaedic Department, Alameda Professor Hernani Monteiro, 4200-319 Porto, Portugal; ^7^Centro Hospitalar de São João, Clinical Pathology Department, Alameda Professor Hernani Monteiro, 4200-319 Porto, Portugal; ^8^Emergency and Intensive Care Department, Centro Hospitalar Sao Joao, Alameda Professor Hernani Monteiro, 4200-319 Porto, Portugal; ^9^Faculty of Medicine, University of Porto, Portugal

## Abstract

*Introduction*. Severity and outcome assessments are crucial in trauma. Our aim was to describe the role of a group of cytokines (TNF*α*, IL-6, IL-10, and HMGB-1) and ICAM-1 as severity and outcome assessment tools and their kinetics in the first 72 h after severe trauma.* Materials and Methods*. Authors designed a prospective cohort study of severe polytrauma patients (ISS > 15) in a level 1 Trauma Centre. Cytokines and ICAM-1 levels and Th1/Th2 ratios were assessed at admission, 24, 48, and 72 h. SIRS, SIRS with hypoperfusion, and shock were identified. Outcomes considered were ICU admission, ARDS, MODS, and death.* Results*. Ninety-nine patients were enrolled (median ISS: 29 and age 31). There was an early release of pro- and anti-inflammatory mediators with higher values at admission (except for ICAM-1). On admission, IL-6 was associated with ISS, IL-10 with SIRS with hypoperfusion, and HMGB-1 with shock. Several cytokines were associated with outcomes, especially IL-6 and IL-10 at 72 h with MODS and death. Low TNF*α*/IL-10 and IL-6/IL-10 ratios at 24 and 72 h were associated with MODS and death.* Conclusions*. Pro- and anti-inflammatory responses occur simultaneously and earlier after injury. Cytokines may be useful for outcome assessment, especially IL-6 and IL-10. Low Th1/Th2 ratio at 24 to 72 h is associated with MODS and death.

## 1. Introduction

Mortality and disability associated with severe trauma are still a significant socioeconomic problem in developed countries [1]. Whilst primary mortality is linked to the initial injuries, particularly with severe trauma brain injury (TBI), haemorrhagic shock, and early complications such as hypothermia, acidosis, and coagulopathy (the triad of death) [2], secondary mortality is strongly related to the development of systemic inflammatory response syndrome (SIRS) and eventually multiple organ dysfunction syndrome (MODS) [3].

Severe trauma induces several neuroendocrine, metabolic, and immunologic changes. The understanding of this changes and its influence on SIRS pathophysiology, including the compensatory anti-inflammatory response syndrome (CARS), as physiological responses to different types of events (infection, burn, trauma, and surgery, as well as others), allowed a better understanding of the onset of severe complications. The model suggested by* Bone RC*, consisting of two distinct phases (an initial proinflammatory that switches to a later anti-inflammatory) was widely accepted [4]. Recently, Xiao and coworkers proposed a new paradigm for the immunological response to severe injury, based on a genomic storm with increased expression of genes in critically injured patients. They suggest that large changes in leukocyte genomic gene expression occur in the first 12 h after injury and are sustained for days or weeks; this early response occurs simultaneously for genes involved in innate and adaptive immune response. The longer and greater the magnitude of these alterations is, the more marked the immunological dysregulation is and the more the patient is prone to severe complications [5]. According to these authors, pro- and anti-inflammatory phases occur simultaneously and not sequentially and the phenotype of trauma-induced immunosuppression may not be fully manifested until days after injury.

As in other models, the outcome in severe trauma is clearly related to the SIRS and CARS progression to MODS. This process depends on a complex network of cellular elements and mediators, including proinflammatory (Th1) and anti-inflammatory (Th2) cytokines and adhesion molecules. In spite of some contradictory results, there is a consensus that the physiopathological process of endothelial injury, which leads to progressive organ failure, is strongly related not only to the initial injuries but also to the level and timing of production and release of these systemic inflammatory response mediators [6, 7]. We hypothesised that the measurement of some of these mediators in the early phase of trauma would help stratify the severity and could be associated with the outcome. We decided to study two predominantly proinflammatory cytokines (TNF*α* and IL-6), one anti-inflammatory cytokine (IL-10), a high mobility protein that acts as a proinflammatory molecule, but also as starter of cellular repair (HMGB-1), and an adhesion molecule closely related to endotheliitis (ICAM-1). Cytokines produced in SIRS may effect Th subset predominance and subsequent immune responses [8], namely, with predominance of early distributive shock (Th1 pattern) or protracted immunoparalysis (Th2 predominance), potentially with increased susceptibility to nosocomial infection. Therefore, we decided to assess Th1/Th2 balance and correlate it to severity and outcome variables.

The goal of our study was to describe the role of a group of cytokines and one adhesion molecule (TNF*α*, IL-6, IL-10, HMGB-1, and ICAM-1) as severity and outcome assessment tools in severe trauma. The description of their kinetics in the first 72 hours and their correlation with the existence of SIRS in the first 6 hours after severe trauma are secondary goals.

## 2. Materials and Methods

### 2.1. Study Design

A prospective cohort study was carried out from January 2010 to December 2010 at Centro Hospitalar São João, Level 1 Trauma Centre, in the North of Portugal. The hospital uses and follows a specific emergency department protocol for severe trauma patients, based on international recommendations. All consecutive adults admitted to the Trauma Room with severe polytrauma satisfying inclusion criteria were enrolled. Inclusion criteria were polytrauma, injury severity score (ISS) > 15, and age > 18 and <65 years. Exclusion criteria were death in the Trauma Room, accident-admission period longer than 360′, noncompliance with the emergency department protocol for severe trauma patients, and transference to a level 2 trauma centre. Ethical approval for this work was obtained through local authority.

### 2.2. Collected Data, Definitions, and Outcomes

Demographic, clinical, imaging, and analytical parameters (at admission, 24, 48, and 72 hours) were obtained from the hospital clinical reports and recorded, at discharge, in a database. The initial injuries at admission were classified by ISS, based on the Abbreviated Injury Scale (AIS), always by the same investigator. The parameters necessary for the diagnosis of SIRS and SIRS with hypoperfusion (SIRS with an increase of lactate or at least one organ dysfunction as a result of the trauma) and shock (SIRS associated with hypotension refractory to fluid therapy and need of vasopressor support) were recorded. The criteria of SIRS, adult respiratory distress syndrome (ARDS) and MODS used were those proposed by the consensual conference between the American College of Chest Physicians and the Society of Critical Care Medicine in 1992 [9].

The negative outcomes considered were intensive care unit (ICU) admission, development of ARDS, and development of MODS and death.

### 2.3. Inflammatory Mediators and Adhesion Molecules Assay

Blood samples were taken at admission and 24, 48, and 72 hours later to measure serum levels of TNF*α*, IL-6, IL-10, ICAM-1, and HMGB-1. Assays were carried out by the same investigator, using the Elisa method, following technical recommendations of Biosource for TNF*α*, IL-6, and IL-10; R&D Systems for ICAM-1; and Shino-Test Corporation for HMGB-1.

### 2.4. Statistical Analysis

Serum levels of the mediators were recorded to describe their kinetics during the first 72 h after severe trauma. Association with the ISS was studied to determine its capacity to assess severity. The behaviour of the mediators and its association with the outcomes were also analysed. With the same goal, the TNF*α*/IL-10 and IL-6/IL-10 ratios were calculated in the four time-points of the study, to assess the Th1/Th2 balance. The correlation between the mediator's levels at admission and the presence of SIRS and SIRS with hypoperfusion and shock were studied.

Statistical analysis was carried out in SPSS v.20.0. Categorical variables were described as absolute and relative frequencies and continued variables by the median and as percentiles, minimums, and maximums. To test the hypotheses about categorical variables independence, Chi-square test or Fisher's exact test was applied. To test continuous variables with nonnormal distribution, Mann-Whitney test was used. Relationships between variables were assessed with Spearman's correlation coefficient. To define cut-off points in each mediator level that predict the outcomes, receiver operator characteristics (ROC) curves were constructed. The area under each ROC and 95% CI were calculated to assess discriminative power. The level of significance was *P* < 0.05.

## 3. Results

During the study period, 99 patients meeting inclusion criteria were enrolled. Almost 3/4 of the patients presented SIRS at admission and 17% were in shock. Concerning the outcomes, ICU admission occurred in 66% of the patients and ARDS developed in 19% and MODS developed in 34% and 28% of the patients died (Table 1).

**Figure 1:**
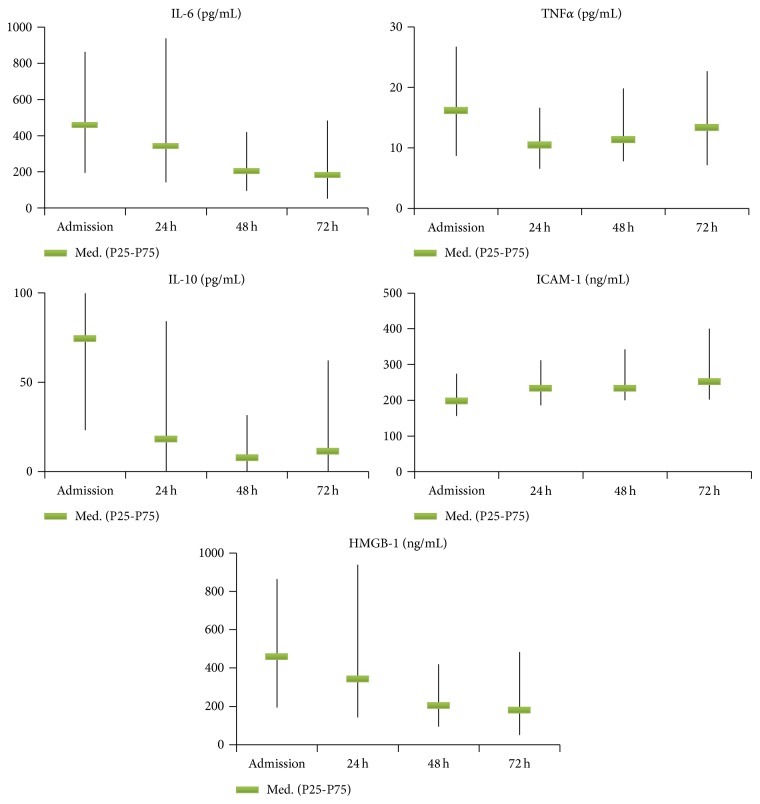
Levels of cytokines, ICAM-1, and HMGB-1 on admission and at 24, 48, and 72 hours (*n* = 99).

**Figure 2:**
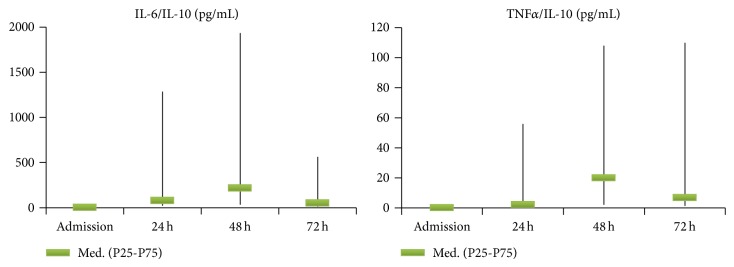
TNF*α*/IL-10 and Il-6/IL-10 ratios on admission and at 24, 48, and 72 h.

Cytokine and adhesion molecule kinetics is depicted in Figure 1. Median TNF*α* at admission was 16.2 pg/mL, maintaining increased levels during the 72 h. For IL-6, higher values (459.5 pg/mL) were found at admission, with a progressive decrease along the 72 h. Median IL-10 showed the highest level at admission (74.45 pg/mL), descending sharply at 24 h. Median ICAM-1 at admission was 198 ng/mL, exhibiting a progressive discreet rise, reaching 252 ng/mL at 72 h. Median HMGB-1 was 10.3 ng/mL at admission, decreasing to around one-third of this value at 24 h. TNF*α*/IL-10 and IL-6/IL-10 ratios evolution are presented in Figure 2.

Concerning severity assessment, ISS was strongly correlated with all negative outcomes (Table 2), but IL-6 was the only mediator correlated, at admission, with ISS (*r* = 0,346). The existence of SIRS at admission was not correlated with any of the adverse outcomes. However, both SIRS with hypoperfusion and shock at admission were correlated with ICU admission and death, but not with ARDS or MODS development (Table 2). Regarding cytokines, ICAM-1 was correlated with the existence of SIRS, IL-10 with SIRS with hypoperfusion, and HMGB-1 with shock (Table 3).

Table 4 shows the association of the mediators' levels and the outcomes studied, depicting those for which the association had statistical significance, using Mann-Whitney test. No cytokine levels on admission were associated with ICU admission. However, IL-6 levels at 24, 48, and 72 h and IL-10 and ICAM-1 levels at 72 h were associated with this outcome. Development of ARDS was associated with IL-10 level at admission at 24, 48, and 72 h and ICAM-1 level at 48 h and IL-6 level at 72 h. MODS development was associated with ICAM-1 level at 24, 48, and 72 h, IL-10 level at 24 and 72 h, IL-6 level at 48 and 72 h, and TNF*α* level at 48 h. Death was associated with IL-10 level at 48 and 72 h and with IL-6 level at 72 h.

The ROC curves and AUC showed interesting cut-off points for IL-6 and IL-10 at 72 hours, associated with MODS and death. IL-6 value >294 pg/mL discriminates MODS (AUC: 0,769; 0,414–0,736) and a value >276 pg/mL discriminates death (AUC: 0,775; 0,591–0,960). IL-10 value >4,93 pg/mL discriminates MODS (AUC: 0,700; 0,506–0,841) and a value >8,24 pg/mL strongly discriminates death (AUC: 0,871; 0,715–1,000).

The relation Th1/Th2 in the four time-points was studied by the ratios TNF*α*/IL-10 and IL-6/IL-10. Those for which the association had statistical significance, using Mann-Whitney test are shown in Table 5. At 24 hours, TNF*α*/IL-10 was associated with ARDS and MODS development and with death and IL-6/IL-10 with ARDS and MODS development. At 72 hours, TNF*α*/IL-10 was associated with ICU admission and death and IL-6/IL-10 with MODS development and death.

## 4. Discussion

In this group of severe trauma patients, several cytokine measurements during the first 72 hours after trauma were associated with several negative outcomes, namely, IL-6 and IL-10 at 72 h with MODS development and death. Low TNF*α*/IL-10 and IL-6/IL-10 ratios at 24 hours and at 72 hours were associated with negative outcomes, namely, MODS development and death. In fact, there was an early release of pro- and anti-inflammatory cytokines (TNF*α*, IL-6, IL-10, and HMGB-1) and adhesion molecule (ICAM-1), the highest values occurring on admission, for all biomarkers except ICAM-1. On admission, IL-6 was associated with ISS, IL-10 with SIRS with hypoperfusion, and HMGB-1 with shock.

The inflammatory mediators and adhesion molecules are decisive in the physiopathology of trauma systemic inflammatory process. TNF*α* is a proinflammatory mediator. It appears in circulation very early (first 1-2 h), being fundamental in the activation of SIRS. It has a half-life of approximately 20 min disappearing from circulation in around 6 hours, which might reduce its role in severity assessment with contradictory results reported in the literature [6]. IL-6 has mainly not only pro- but also anti-inflammatory actions and an important role in endothelial adhesion [10]. It appears early in circulation after trauma (1–4 hours), remaining for a few days [6]. HMGB-1 is a structural nuclear protein linked to the DNA, promoting the recruitment of mononuclear cells and starting the process of cellular repair [11] and acting as an indicator of necrosis and as a proinflammatory cytokine in the processes of ischemia, burn, sepsis, trauma, oncological diseases, and inflammatory illnesses [12]. IL-10 has an important function in the anti-inflammatory phase, being traditionally considered as a late mediator associated with CARS. ICAM-1 is an adhesion molecule associated with endotheliitis and plays an important role in the physiopathological process of SIRS-MODS [13]. This overwhelming systemic inflammation may lead to cellular oedema hypoxia death, with consequent parenchymal lesion and progressive organ dysfunction (MODS) [14]. The classical paradigm of an initial proinflammatory response that switches later to anti-inflammatory response, as proposed by Bone [4], seems to be contradicted by kinetics of the mediators in our study, much more supporting Xiao's model [5]. In fact, the highest values of both Th1 and Th2 cytokines were found at admission, few hours after injury. Actually, IL-10 levels, a Th2 cytokine, decreased markedly in the first 24 hours. Only the adhesion molecule ICAM-1 showed a slightly creeping curve along the 72 hours.

In our study, SIRS with hypoperfusion and shock were associated with ICU admission and death, as previously described [15], while SIRS without hypoperfusion or shock did not, probably for being too unspecific as a marker of severity. ICAM-1, IL-10, and HMGB-1 levels at admission were associated, respectively, with SIRS and SIRS with hypoperfusion and shock. The association of HMGB-1 with shock in trauma confirms results obtained by Cohen et al. [7], although in their study the association extended also to SIRS and SIRS with hypoperfusion.

Severity assessment in trauma is classically based on anatomical scores, namely, the ISS [16], combined or not with physiological variables and age. However, anatomical damage seems to be associated with more marked systemic inflammation and endotheliitis, as shown by the association between IL-6 levels at admission and ISS in our study and other studies [17, 18]. Finally, systemic inflammation is a driver of organ dysfunction and worse outcome.

In our study, all inflammatory mediators tested, except for TNF*α* and HMGB-1, were associated with several negative outcomes, as ICU admission and ARDS and MODS development or death. However, at admission, only IL-10 was associated with one of the outcomes, namely, ARDS development. TNF*α* early disappearance might reduce its importance for severity or outcome assessment, with contradictory results reported in the literature [6] and, in our study, its discriminative power was negligible. The strongest associations, in our study, were shown by IL-6 and IL-10 at 48 and 72 h for ARDS or MODS development or death. The associations of IL-6 and IL-10 levels at 72 h with mortality were particularly relevant. In previous studies, IL-6 levels above 500 pg/mL had been shown to be related to MODS and death [19]. In our study, at least one of the measurements of IL-6 in the first 72 hours was associated with all the outcomes considered and IL-6 at 72 hours was associated with four of them. The associations between IL-6 at 72 hours and MODS development (AUC = 0,769 for a cut-off > 294 pg/mL) and death (AUC = 0,775 for a cut-off > 276 pg/mL) were especially relevant. An increase of IL-10 in serum and in alveolar lavage was reported as being associated with ARDS in trauma [20, 21] and our study shows that IL-10 levels at admission and at 24, 48, and 72 hours were associated with the development of ARDS. Our study also confirms its association with outcome, suggested by others [22], as IL-10 levels at 72 hours were correlated with MODS development (AUC = 0,700 for a cut-off level > 4,93 pg/mL) and especially with death (AUC = 0,871 for a cut-off level > 8,24 pg/mL). ICAM-1, as IL-6, is a marker of endothelial inflammation and dysregulation and its levels have also been shown, in previous studies, to be correlated with the severity of the lesions and with MODS [17, 23]. In our study, ICAM-1 levels at 24, 48, and 72 hours were associated with MODS development but not with death and the association was much weaker than that for IL-6 and IL-10 at 72 hours. The fact that the strongest correlations occurred at 72 hours may be a marker of the importance of therapy in the first couple of days after trauma for the outcome and also suggest that the persistence of raised levels of pro- and anti-inflammatory cytokines may be a driver, or at least a marker, of progressive and irreversible organ dysfunction. Our study also shows that having lower Th1 ratio (TNF*α* or IL-6) than Th2 (IL-10) cytokines ratio, at 24 to 72 hours after trauma, is associated with the worst outcome, namely, ARDS and MODS development and death. This association may be due to the fact that the predominance of Th2 cytokines may lead to immunoparalysis that generates a higher incidence of nosocomial infection, worsening prognosis. Lack of recovery in monocyte human leukocyte antigen-DR expression has been shown to be associated with the development of sepsis after trauma [24]. Therefore, assessment of inflammatory response in early severe trauma should be included in studies regarding early stratification of outcome, allowing a more solid decision regarding ICU admission and the identification of a population of trauma patients prone to the acquisition of nosocomial infection. In the near future, modulation of Th cell subset predominance may become a novel therapeutic option in the treatment of severe trauma.

Authors recognise some limitations, namely, the small sample size, with the fact that it is a single centre study and the absence of data on nosocomial infection.

## 5. Conclusions

Our study suggests that pro- and anti-inflammatory responses occur simultaneously and in an early phase after injury and that cytokine measurement may be useful for outcome assessment, especially IL-6 and IL-10, 48 to 72 hours after trauma. Having a lower Th1 ratio than Th2 ratio at 24 to 72 hours after trauma is associated with the worst outcome, namely, MODS development and mortality.

## Figures and Tables

**Table 1:** Patients demographics, injury mechanism, ISS, SIRS (at admission), and outcomes.

	(*n* = 99)
Age med. (P25–P75)	31 18–60
Male/female (%)	83/17
Injury mechanism	
Traffic	81
Work	6
Other	13
ISS med. (P25–P75)	29 (17–52)
SIRS (%)	73
SIRS with hypoperfusion (%)	39
Shock (%)	17
Outcomes (%)	
UCI admission	66
ARDS	19
MODS	34
Death	28

**Table 2:** Relationship between age, ISS, and SIRS classification on admission and the outcomes (*n* = 99).

		ICU admission		ARDS		MODS		Death	
		Yes	No	Yes	No	Yes	No	Yes	No
Age^*^	Med.	34	22	39	29	32	30	27	31
	P25–P75	18–62	18–54	24–65	18–60	18–62	18–58	18–65	18–58
	*P* ^*^	***0.019***		***0.008***		*0.149 *		*0.750 *	

ISS^*^	Med.	30	22	35	29	30	26	34	28
	P25–P75	19–54	15–38	17–66	17–48	21–59	17–45	25–54	17–45
	*P*	***0.001***		***0.012***		***0.006***		***0.002***	

SIRS (*n* = 73)	*n*	50	23	17	56	26	47	21	52
	%	68	32	76	24	36	64	29	71
	*P* ^**^	*0.518 *		*0.078 *		*0.655 *		***0*** *.858 *	

SIRS with hypoperfusion (*n* = 39)	*n*	33	6	11	27	16	23	17	22
	%	85	15	29	71	41	59	44	56
	*P* ^**^	***0.002***		***0.057***		*0.259 *		***0.006***	

Shock (*n* = 17)	*n*	17	0	6	11	9	8	14	3
	%	100	0	38	62	53	47	82	18
	*P* ^**^	***0.001***		*0.077* ^***^		*0.076 *		***<0.001***	

^*^Mann-Whitney test, ^**^qui-square test (Pearson), and ^***^exact test of Fisher.

**Table 3:** Relationship between mediators' levels and degree of inflammatory response (Mann-Whitney test).

		SIRS		SIRS with hypoperfusion		Shock	
		Yes	No	Yes	No	Yes	No
IL-10 (admission) pg/mL	Med.			122.5	51.9		
	P25–P75			38.4–239	13.2–118		
	*P *			*0.01 *			

IL-10 (24 h) pg/mL	Med.	9.19	0.1	13.3	1.16		
	P25–P75	0.1–23	0.1–2.45	4.34–25.6	0.1–10.3		
	*P *	*0.007 *		*0.002 *			

ICAM-1 (admission) ng/mL	Med.	252	180				
	P25–P75	210–338	147–241				
	*P *	*0.04 *					

HMGB-1 (admission) ng/mL	Med.					15.9	8.4
	P25–P75					5.37–30.1	2.6–16
	*P *					*0.047 *	

**Table 4:** Relationship between mediators and the outcomes (Mann-Whitney test).

		Yes Med. (P25–P75)	No Med. (P25–P75)	*P *
ICU admission				
IL-6 pg/mL	24 h	569 (177–1440)	190 (131–439)	*0.006 *
	48 h	315 (140–567)	117 (60–204)	*0.001 *
	72 h	295 (129–585)	53.7 (29.1–171)	*0.001 *
IL-10 pg/mL	72 h	3.35 (0.1–15.2)	0.1 (0.1–4.0)	*0.0019 *
ICAM-1 ng/mL	72 h	272 (206–445)	222 (191–316)	*0.048 *

ARDS				
IL-6 pg/mL	72 h	419 (135–622)	145 (36.4–429)	*0.036 *
IL-10 pg/mL	Admission	128 (102–187)	51.1 (16.4–146)	*0.007 *
	24 h	13.1 (8.3–46.8)	3.6 (0.1–14.3)	*0.003 *
	48 h	6.7 (0.1–23.3)	0.1 (0.1–4.3)	*0.035 *
	72 h	10.8 (1.2–16.3)	0.1 (0.1–5.4)	*0.015 *
ICAM-1 ng/mL	48 h	342 (234–457)	225 (196–311)	*0.031 *

MODS				
IL-6 pg/mL	48 h	319 (242–711)	169 (86–344)	*0.034 *
	72 h	405 (129–1184)	112 (36–271)	*0.002 *
IL-10 pg/mL	24 h	10.2 (3.6–25.6)	1.2 (0.1–14.3)	*0.008 *
	72 h	10.8 (1.2–17.7)	0.1 (0.1–3.6)	*<0.001 *
ICAM-1 ng/mL	24 h	306 (220–370)	202 (178–287)	*0.018 *
	48 h	296 (230–486)	220 (194–300)	*0.034 *
	72 h	307 (200–614)	240 (202–322)	*0.002 *
TNF*α* pg/mL	48 h	17.8 (10.4–24)	10.3 (6.4–16.4)	*0.016 *

Death				
IL-6 pg/mL	72 h	441 (197–1527)	144 (47–445)	*0.024 *
IL-10 pg/mL	48 h	10.9 (0.1–16.8)	0.1 (0.1–4)	*0.025 *
	72 h	19.1 (11.6–40.6)	0.1 (0.1–5.0)	*0.001 *

**Table 5:** Relationship between TNF*α*/IL-10 and IL-6/IL-10 ratios and outcome.

	P25	Med.	P75	P25	Med.	P75	*P* ^*^
**ICU admission**	**No**			** Yes**			
TNF*α*/IL-10 at 72 h	5.18	55.1	136.5	0.88	3.53	98.5	*** 0.038***
**ARDS**	**No**			** Yes**			
TNF*α*/IL-10 at 24 h	0.62	3.14	70.15	0.20	0.78	2.63	*** 0.013***
IL-6/IL-10 at 24 h	33.53	174.07	1360	9.08	22.31	46.7	*** 0.004***
**MODS**	**No**			** Yes**			
TNF*α*/IL-10 at 24 h	0.56	4.20	95.5	0.31	1.22	3.22	*** 0.028***
IL-6/IL-10 at 24 h	33.5	472.59	1420	15.5	45.24	148.3	*** 0.008***
IL-6/IL-10 at 72 h	27.9	126	772	20.1	39.9	94.6	*** 0.002***
**Death**	**No**			** Yes**			
TNF*α*/IL-10 at 24 h	0.69	2.97	66.6	0.22	0.64	4.51	*** 0.028***
TNF*α*/IL-10 at 72 h	2.69	44.45	123	0.46	1.65	2.54	*** 0.002***
IL-6/IL-10 at 72 h	23.3	106.03	1060	12.6	35.66	46.1	*** 0.037***

^*^Mann-Whitney test.
